# Does China’s Carbon Trading Pilot Policy Reduce Carbon Emissions? Empirical Analysis from 285 Cities

**DOI:** 10.3390/ijerph20054421

**Published:** 2023-03-01

**Authors:** Xuehui Yang, Jiaping Zhang, Lehua Bi, Yiming Jiang

**Affiliations:** 1 School of Business, Jinggangshan University, Ji’an 343009, China; 2 School of Public Administration, Faculty of Economics and Management, East China Normal University, Shanghai 200062, China; 3 School of Economics, Guangxi University, Nanning 530004, China; 4 Xingjian School of Science & Liberal Arts, Guangxi University, Nanning 530004, China

**Keywords:** Carbon Trading Pilot Policy, green consumption transformation, ecological efficiency, industrial structure

## Abstract

This article studies the influence of the Carbon Trading Pilot Policy (CTPP) on carbon emissions by constructing the balanced panel data from 2003 to 2020 for 285 cities in China above the prefecture level. Difference-in-Difference (DID) method is used to test the influence and the mechanism. (1) The findings suggested that CTPP has dramatically reduced China’s carbon emissions by 6.21%. The parallel trend test shows that the premise of DID is reliable. (2) A variety of robustness tests, such as the instrumental variable method for endogeneity, Propensity Score Matching (PSM) for sample selection bias, variable substitution, time–bandwidth change, and exclusion of policy intervention, show that the conclusion is still robust. (3) The mediation mechanism test indicates that CTPP can promote the reduction in carbon emissions by promoting Green Consumption Transformation (GCT), improving Ecological Efficiency (EE), and promoting Industrial Structure Upgrading (ISU). GCT contributes the most, followed by EE and ISU. (4) The analysis of the heterogeneity reveals that CTPP has a greater effect on carbon emission reduction in central and peripheral cities in China. This study provides policy implications for China and similar developing countries in the face of carbon reduction.

## 1. Introduction

Scholars tend to agree on the adverse effects of large amounts of carbon emissions on the human environment [1,2]. For example, excess carbon dioxide intensifies the effect of greenhouse, causing global warming, and accelerating the melting of the polar glaciers in both the north and south, resulting in rising sea levels and shrinking human habitats. Accelerating global temperatures could affect the growth of food crops around the world, diminish people’s quality of life and harm people’s physical health.

Since opening to the exterior, China’s economy has experienced sustained, rapid, and consistent growth over the long term, but its carbon emissions have also increased year by year. In 2011, China’s GDP became the world’s second largest, overtaking that of Japan (Source: http://jingji.cntv.cn/20110121/105731.shtml, accessed on 1 November 2022). In 2020, China’s GDP reached 101.36 trillion yuan, surpassing 100 trillion yuan for the first time (Source: https://data.stats.gov.cn/, accessed on 1 November 202). China’s average annual GDP growth rate (1979–2020) was 9.2% (Source: http://sky.cssn.cn/jjx/jjx_xzyc/202209/t20220913_5492367.shtml, accessed on 1 November 2022). The cost behind China’s economic growth “miracle” is exceptionally high. Its economic development excessively depends on the mode of extensive economic development of “high input, high consumption, and high pollution”, which makes China the greatest carbon emitter in the world [3].

In the face of the increasingly prominent carbon emission problem, the Chinese government has actively explored reasonable and feasible solutions. As early as 1979, China’s first law on environmental protection was enacted and implemented—*the Environmental Protection Law* (Source: https://www.chinacourt.org/law/detail/1989/12/id/10120.shtml, accessed on 1 November 2022). In 2003, China promulgated *the Regulations on the Collection and Use of Pollutant Discharge Fees*, emphasizing the implementation of environmental protection subsidies and encouraging enterprises to conduct pollution control in reverse (Source: http://www.gov.cn/zhengce/content/2008-03/28/content_5152.htm, accessed on 1 November 2022). In 2011, China issued the *National Environmental Protection Laws, Regulations, and Environmental Economic Policy Construction Plan during the 12th Five-Year Plan Period* (Source: https://www.mee.gov.cn/gkml/hbb/bwj/201111/t20111109_219755.htm, accessed on 1 November 2022). Chinese President Xi Jinping put forward the target of “Carbon Emissions Peaking and Carbon Neutrality” during the 75th United Nations General Assembly on 9 December 2020.

This article studies the effectiveness and mechanism of China’s Carbon Trading Pilot Policy (CTPP) on carbon emissions. In 2011, China released *the Notice on Implementation of the Carbon Trading Pilot Policy (CTPP)*, which designated Beijing, Shanghai, Tianjin, Chongqing, Hubei, Guangdong, and Shenzhen as pilot carbon trading areas (Source: https://zfxxgk.ndrc.gov.cn/web/iteminfo.jsp?id=1349, accessed on 1 November 2022). These seven cities and provinces have consecutively implemented CTPP since 2013. In 2016, Sichuan and Fujian began to implement the policy. As of the end of 2022, China’s carbon transaction volume has exceeded RMB 9 billion, with the total turnover exceeding 400 million tons (Source: http://www.gov.cn/xinwen/2020-10/28/content_5555655.htm, accessed on 1 November 2022). However, China is one of the developing countries implementing CTPP. Compared with developed countries, China’s carbon market has many deficiencies, such as insufficient competition regulation, and weak political and economic constraints [4,5]. In this sense, exploring China’s CTPP has important reference significance for other similar developing countries [6].

CTPP is a market-based environmental policy, distinct from the previous command-based environmental policy. Market-type environmental policy is mainly market-oriented, guiding enterprises to adjust their economic activities and effectively reducing environmental pollution [7]. Environmental policy is a crucial component of the social and environmental governance system [8], an essential tool for building an environment-friendly society. The Chinese government often explores appropriate policy tools through policy pilots, and then promotes them nationwide [9]. The same applies to CTPP. Remarkable progress has been made in China’s carbon emissions. However, is there a significant intrinsic link between China’s CTPP and carbon emissions? What is the transmission mechanism? Previous studies have been controversial, so it is urgent to analyze and test the effect of China’s CTPP.

**Figure 1:**
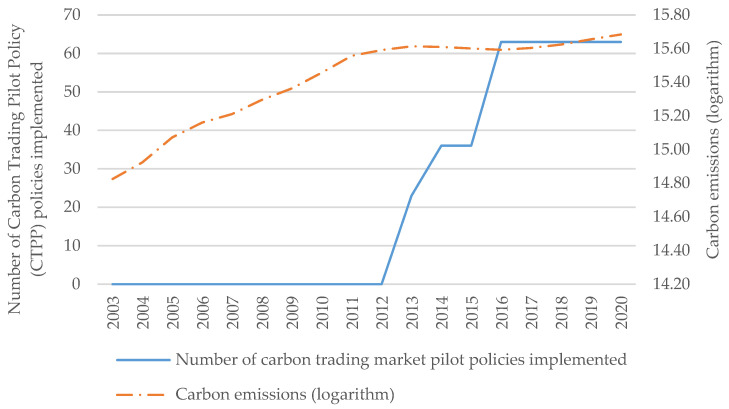
Number of cities in China implementing the Carbon Trading Pilot Policy (CTPP) and national carbon emissions (Source: Authors’ construct).

We plot the connection between the number of cities implementing CTPP and carbon emissions in China from 2003 to 2020 (see Figure 1). From 2003 to 2020, with the increase in the number of cities implementing CTPP, the growth acceleration of China’s carbon emissions shows a trend of gradual slowdown. This trend suggests that CTPP may be effective. Of course, to further determine the connection between CTPP and carbon emissions, we are going to conduct an empirical test through econometrics.

The following three areas will be the highlights and novel aspects of this article. First, CTPP is used as a quasi-natural test. The Difference-in-Difference model (DID) is utilized to investigate the relationship between market-based carbon trading policies and carbon emissions. Different from provincial panel data in most of the previous literature, this paper uses city panel data for empirical analysis and testing. This helps to obtain more precise conclusions. Second, the mechanism analysis and empirical test are carried out in depth. From the standpoint of Green Consumption Transformation (GCT), Ecological Efficiency (EE), and Industrial Structure Upgrading (ISU) investigate the effect of environmental policies on carbon emissions and additionally investigate the relative contributions of various mechanisms to the process of reducing carbon emissions. Third, the heterogeneity of CTPP is discussed from regional and city perspectives, which helps propose targeted policy suggestions.

Below is the remaining content from the article. The second section is a literature review, which reviews the previous relevant literature, and points out the shortcomings of the research and the innovation points of this paper. The third section is the mechanism analysis, which puts forward three hypotheses. Data sources and methods are introduced in the fourth part, introducing the data source and the DID method. The fifth section is the baseline regression results, testing the intermediary mechanism of CTPP affecting carbon emissions. The sixth part is the robustness test, including endogeneity treatment, Propensity Score Matching (PSM), variable substitution, one-period lag, changing the time–bandwidth, and excluding other policy disturbances. The seventh part tests the theoretical mechanism. The eighth section is devoted to some heterogeneity analysis. The final section presents brief conclusions and policy implications.

## 2. The Literature Review

At present, many scholars and policymakers attach great importance to the issue of carbon emissions. Scholars have made some rich academic achievements in research topics related to the carbon trading market. This paper summarizes the economic and social benefits, emission reduction effect, mechanism path, and other aspects of carbon trading, as follows.

First, scholars evaluated the economic and social benefits of low-carbon policies. For example, Du and Wang (2011) [10] constructed a low-carbon city evaluation index system and evaluated the low-carbon city construction. Yang and Li (2013) [11] evaluated the progress of the first eight pilot low-carbon cities in China and proposed specific requirements for the construction of low-carbon cities. Some scholars believe that the carbon trading system can maximize the cost benefit and generate considerable economic benefits [12], increase the industrial output value by 13.6% [13], and promote the transformation of the low-carbon economy [14]. In addition, it can improve energy efficiency [15], promote industrial structure upgrading [16], promote enterprise technological innovation [17] and green and low-carbon innovation [18,19], promoting high-quality development of the manufacturing industry [20], enhancing low-carbon international competition of the industry [21] and promoting technological maturity of exports [22]. In addition, CTPP will reduce the level of government green investment due to the substitution effect (Han, 2020) [23], and so on.

Second, we examined the carbon emission reduction effect of the carbon emission trading system. Scholars from the provincial, prefecture, city, industry, and enterprise levels, respectively, have carried out a relatively sufficient study. Zhang and Shi et al. (2017) [24] adopted the simulation analysis of China’s provincial panel data and proved that the implementation of carbon emission trading in China can significantly save energy and reduce emissions, which can reduce carbon intensity by 20.06%. Xia and Li et al. (2020) [25] used provincial data in China and determined that China’s carbon emission trading system would reduce at least 4 million tons of carbon emissions every year. Some scholars constructed panel data of prefecture-level cities and determined that carbon trading systems effectively reduced carbon emissions [26] and carbon emission intensity per unit GDP [27]. In addition, scholars study enterprises such as industry [28] and listed companies [29,30]. They discovered the carbon emission reduction effect of the carbon emission trading system. However, some scholars believe that CTPP will lead to carbon leakage, that is, transfer from non-pilot areas to pilot areas through market participation and industrial transfer [31].

Third, we examined the research on the carbon emission reduction mechanism of CTPP. The literature in this area is not sufficient, and the research conclusions are quite diverse. Based on the correlation between Beijing’s industrial structure adjustment and emissions of carbon dioxide, Mi et al. discovered that the former significantly affects the latter [32]. Nevertheless, Zhang et al. analyzed data from China’s 281 prefecture-level cities from 2006 to 2016 using a dynamic spatial panel model [33]. They discovered that improving industrial structure insignificantly affects the intensity of carbon emissions (CEI). Hong and Cui et al. (2022) [15] concluded that China’s carbon trading system could improve cities’ total factor energy efficiency through green innovation and resource allocation. Wang and Huang et al. (2022) [34], from the perspectives of economy, politics, culture, society, and ecological civilization, concluded that CTPP could reduce carbon emissions through industrial structure adjustment, low-carbon policy coordination, cultural communication, green space construction, energy intensity reduction, and other aspects. Regarding the mechanism of carbon emission reduction, scholars have different opinions. Some believe that the carbon-trading mechanism promotes carbon emission reduction through energy consumption structure [35] rather than industrial structure [36]. Some scholars also believe that carbon quota price and the number of enterprises participating in carbon trading are key factors affecting carbon emission reduction [37]. Chen and Shi et al. (2020) [38] constructed provincial panel data and determined that CTPP reduces carbon emissions through technical, structural, and configuration effects.

Fourth, we studied the link between environmental policy and carbon emissions. Prior research primarily examined environmental policies that were based on market forces and orders from above. Scholars have studied different environmental policy instruments and reached different conclusions. For example, Blackman and Kildegaard studied the effectiveness of mandatory environmental regulation policies in Mexico. They theorized that those environmental policies did not effectively stimulate the green technology innovation of the enterprises, but increased their pollution emissions [39]. Zheng and Shi studied China’s environmental regulation policies and determined that pollution reduction targets had not been achieved [40]. Wang et al. concluded that CTPP could not reduce sulfur dioxide emissions [41]. In contrast to this view, Marconi examined the effect of China’s and the EU’s mandated environmental regulations on the emissions of pollution-intensive businesses and discovered that these regulations had a favorable influence on lowering carbon emissions [42]. According to Cheng et al., a market-based emissions trading system will have a negative effect on Guangdong Province’s carbon emissions, which are expected to fall to two-thirds of their 2010 levels by 2020 [43]. Liao et al. used the Shanghai CTPP as an illustration and discovered that the application of environmental policies greatly affects the decrease in regional carbon emissions [44].

To sum up, emissions of carbon have been extensively examined in the past literature. Previous studies have comprehensively elaborated on the characteristics and operating mechanisms of the carbon trading market. However, there are shortcomings, as follows. First, in the space and time range of the previous studies, the scholars mainly focused on provincial or industrial carbon emission reduction effects [14,18,45], and there is a lack of nationwide environmental policy testing. The findings are inconsistent and controversial, especially regarding the transmission mechanism. Second, the current research focuses on carbon emission reduction, but the empirical methods ignore the parallel trend test and endogeneity treatment, and so on. These may reduce the reliability of the conclusions. Third, the previous focus of the relevant literature studies is only on testing the influence of environmental regulating policies on the decrease in carbon emissions and analysis of mechanisms, but not on the measurement of the contribution of various mechanisms to carbon emission reduction. Finally, a lack of literature exists on the urban grade heterogeneity of environmental regulation policies, and a vast bulk of the literature centers on the repercussions of environmental regulating laws on lowering carbon emissions.

Compared with previous studies, this paper has certain uniqueness. First, this article revisits the repercussions of environmental policies on carbon emissions from another angle. The previous literature has examined chiefly the impacts of command-based environmental policies. Therefore, this article is founded on the pilot policy of market-based carbon trading (CTPP). Second, this article researches both the policy influence and the theoretical mechanism. From the standpoint of green consumption, industrial structure, and ecological efficiency, this article investigates the connection between the CTPP and carbon emissions, which is rarely seen in the previous studies and would enrich the relevant body of literature. Third, endogenous problems, such as reverse causality and variable omission, are tested by the instrumental variable method, and the probability of selection bias is dealt with by the Propensity Score Matching (PSM) method. The paper enriched the body of the literature related to empirical detection. Fourth, we test not only the mechanisms but also the contribution of each mechanism.

## 3. Theoretical Mechanism Analysis and Hypotheses

Unlike previous command-based environmental policies, we study the impact of market-based environmental policies, namely the Carbon Trading Pilot Policy (CTPP), on carbon emissions. We believe that market-based environmental policies indirectly promote the lowering of carbon emissions mainly through three effects: Green Consumption Transformation (GCT) effect, Ecological Efficiency (EE) effect, and the Industrial Structure Upgrading (ISU) effect. The three specific theoretical mechanisms are analyzed as follows.

### 3.1. Green Consumption Transformation Effect

Environmental regulation policies can help promote Green Consumption Transformation (GCT). For the implementation of any policy, the relevant departments of the Chinese government and the media will publicize and explain it to the public, and the CTPP is the same. From one perspective, the repercussions of environmental policies will be good for consumers’ environmental knowledge, environmental awareness, social norms, and environmental behaviors [46], thus improving consumers’ environmental responsibility and environmental attention and promoting green consumption [47]. From another perspective, environmental policies will raise energy prices. According to the “Green Paradox” [48], in the short term, it will encourage consumers to increase non-green consumption significantly; however, still, in the long run, the proportion of green consumption will significantly increase.

The green consumption transition helps reduce carbon emissions. Switching to greener consumption usually reduces energy consumption and CO_2_ emissions [49]. The green consumption model replaces the original high-carbon consumption model, changes the consumption tendency, increases the consumption proportion of low-carbon goods, chooses more environmentally friendly goods, and reduces carbon emissions. The transformation of consumption patterns to green offers an essential contribution to reducing carbon dioxide emissions [50]. In addition, the insufficient quantity and the low quality of green supplies often lead to the low willingness of consumers to consume green products. Given such problems, producers must effectively integrate environmental issues into their production process to reduce the environmental impact [51] and provide more high-quality green supplies to meet the consumers’ demand. Promoting the transformation of green consumption, rationally expanding the demand for green supplies, and following the concept of green consumption are all conducive to reducing carbon emissions. To sum up, this paper advances Hypothesis 1.

**Hypothesis** **1:** *CTPP reduces carbon emissions by promoting Green Consumption Transformation (GCT).*

### 3.2. Ecological Efficiency Effect

Environmental policies are helpful in improving ecological efficiency. Environmental policies have considerably reduced the amount of urban sewage discharge and achieved the goal of improving urban ecological efficiency [52]. Environmental regulations that are reasonable and rigorous can promote economic innovation, advance production technology, and raise total factor productivity (TFP) [53], thus offsetting the costs caused by environmental governance [54] and so enhancing ecological efficiency. By playing the role of the government as a “bellwether” and taking technological innovation as a critical path, environmental policies encourage the widespread use of low-carbon technologies, as well as provide guarantees for improving ecological efficiency [55].

Carbon emissions are accompanied by pollutant emissions. The improvement of ecological efficiency requires strict restrictions on high-pollution, high-energy-use, and high-emission industries, optimization of resource allocation, increase in the input of production factors with low carbon and low energy consumption, production of greener products, and reduction in pollution emissions, eventually reducing carbon emissions. Promoting sustainable clean production, increasing the circular economy, reducing carbon emissions, and conserving energy are all facilitated by increasing ecological efficiency [56]. One of the key elements to encourage the decrease in carbon emissions is improving ecological efficiency. Therefore, the following Hypothesis 2 is proposed.

**Hypothesis** **2:** *CTPP reduces carbon emissions reduction by improving Ecological Efficiency (EE).*

### 3.3. Industrial Structure Upgrading

Environmental policies are conducive to promoting the upgrading of the industrial structure. The CTPP offers companies a certain amount of carbon emission rights. If the firms exceed their emissions, they need to purchase them at additoional costs.

According to the “environmental compliance cost hypothesis,” when the government imposes strict environmental regulations, industries with high consumption and pollutant levels will relocate to regions with lax environmental regulations. At the same time, the impact on cleaner industries will be minimal. This will force an upgrade in the industrial structure [57]. The secondary industry’s share of GDP declines as a result of environmental legislation [58]. Environmental performance and the number of secondary industries are significantly inversely correlated [59]. The decrease in pollutant emissions and carbon emissions can be achieved by encouraging the transfer and upgrading of industries.

In addition, according to the enterprise “Innovation Compensation Theory” [60] or “Pollution Refuge Hypothesis” [40], when governments enforce a strict regulatory environment, high costs of production and high-pollution industries will increase and the profit margins of enterprises will be reduced. In order to change this unfavorable situation, enterprises may reduce or stop their energy-intensive production activities or shift to cleaner industries, thus promoting industrial structure upgrading.

The traditional extensive production mode has changed due to the modernization of the industrial structure, which has helped the transition to low-carbon production [61]. Low-carbon production can effectively lower energy consumption and carbon emissions and foster economic development. There is unidirectional G-Causality between industrial structure improvement and carbon emission [62]. Modernizing industrial structures offers enormous potential to cut carbon emissions [32]. Reducing carbon emissions will be made possible by modernizing the industrial structure, advancing technology, and increasing environmentally friendly and low-carbon manufacturing methods [63]. Therefore, this research puts forward hypothesis 3.

**Hypothesis** **3:** *CTPP can lower carbon emissions by encouraging Industrial Structure Upgrading (ISU).*

**Figure 2:**
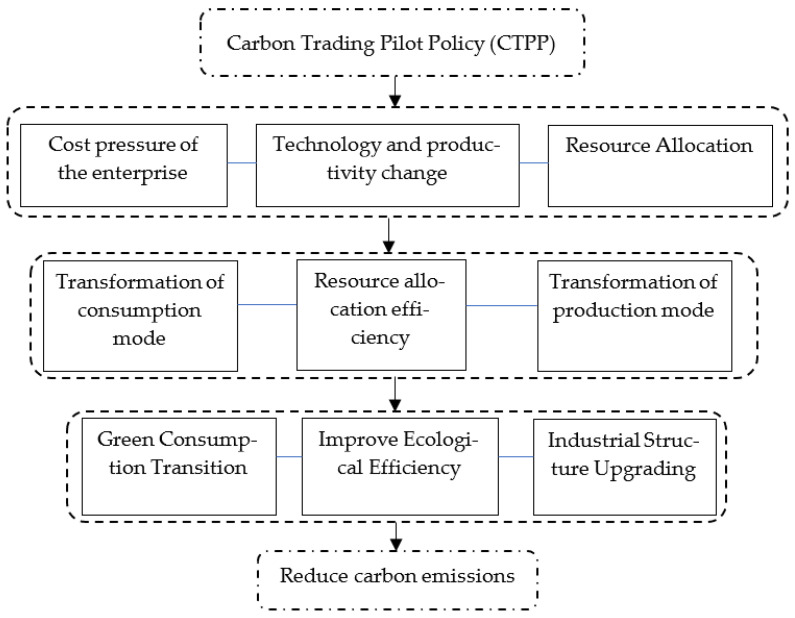
Action mechanism of environmental regulation and carbon emission reduction. (Source: Authors’ construct).

Based on the above analysis, the three mechanisms by which CTPP reduces carbon emissions are presented in Figure 2.

## 4. Data and Methods

### 4.1. Data

This article covers 285 prefecture-level cities in China, including those directly administered by the Central Government, due to the absence of data in Tibet and the adjustment of the administrative regions of Chaohu City and other cities. The sample period was from 2003 to 2020, with 5130 samples. The carbon emission data was obtained from the website of “the Center for Global Environmental Research”, utilizing the compiled and collected data on prefecture-level cities’ carbon emissions from 2003 to 2019. The data for 2020 were obtained by insertion method. The Carbon Trading Pilot Policy (CTPP) information was obtained from the official government websites of each province or city by manual sorting. Data on the instrumental variables were obtained from the ERA-Interim database of the European Centre for Medium Weather Forecasts (ECMWF). Additional data were gathered from the China City Statistical Yearbook (2004–2021). The missing data were supplemented by linear insertion, and the data indexes were sorted and calculated.

### 4.2. Variables Description

(1) Explained variable

Carbon Emissions (*ln_co_2_*). This article selects the emissions of carbon dioxide of each city over the years as the explained variable (logarithm).

(2) Core explanatory variable

Carbon Trading Pilot Policy (CTPP). CTPP is denoted by 0 or 1, where 1 indicates policy implementation and 0 otherwise. According to the official website of the provincial or city government, CTPP has been implemented in 2013. In 2016, Sichuan and Fujian provinces also adopted the policy.

(3) Control variables

Level of Opening-up (*ln_fdigdp*). The amount of opening-up is gauged using the GDP to foreign direct investment (FDI) ratio. What effect does FDI have on carbon emissions? Academics hold different opinions. Baek argued that foreign direct investment tends to deteriorate the environment in the long term and the short term [64], thus becoming a “pollution paradise” for foreign investors [65]. Additionally, some academics theorize that FDI increases the host nation’s carbon dioxide emissions [66]. However, Wang and Jing determined that foreign direct investment would bring advanced technology, improve the environmental quality of the investment place and have a “spillover effect” [67].

Investment in Science and Technology (*ln_sciep*). Science and technology are crucial to reducing carbon emissions. Technology advancements are contributing to lower carbon emissions and an increase in energy efficiency [68]. In our work, the investment in science and technology is represented by the per capita fiscal expenditure on these fields.

Education Input (*ln_edue*). Education affects economic development and technological innovation [69]. Higher education levels may be associated with greater environmental awareness. This paper uses the education expenditure of each city to represent the Education Input.

Economic Status (*ln_gdppop*). The per capita GDP of each city represents the economic Status. Numerous connections between economic levels and carbon emissions have been uncovered in existing studies. The linkage between environmental quality and economic growth is depicted as an inverted U-shaped trend, as seen by the EKC. Existing research, however, points to further themes, including inverted “U” shapes, positive “U” shapes, inverted “N” shapes, and others. In addition, Aye and Edoja found that carbon emissions are related to economic growth speeds, with significant positive effects at low growth speeds and higher marginal effects at high speeds [70].

Information Status (*ln_internetp*). Information and Communication Technology (ICT) infrastructure is critical to driving green development [71]. To limit the effect of information technology status on carbon emissions, internet users per 10,000 individuals in each city are employed in this study to represent information status. Aiming to regulate the impact of information technology on carbon emissions, our article quantifies the information level using the ratio of internet users per 10,000 people.

Industrial Structure (*ln_secgdp*). The structure of the industrial sector affects carbon emissions. The secondary industry is where the majority of carbon dioxide emissions are produced. Additionally, the tertiary industry’s increased production value helps to cut down on carbon emissions [72] Therefore, the industrial structure was measured in our work using the logarithm of the secondary industry’s share of GDP.

Population Density (*ln_popden*). Population density positively correlates with environmental pollution [73]. The index is expressed as a ratio of the population to the administrative area.

Energy Consumption. Carbon emissions and energy use generally have a positive relationship. According to the method of Yang et al., Per Capita Natural Gas Supply (*ln_gasp*), Per Capita Liquefied Gas Supply (*ln_liqgasp*), and Per Capita Electricity Consumption (*ln_elecp*) represent energy consumption [74].

Variable definitions and descriptive statistics are presented in Table 1.

### 4.3. Method

We used the Difference-in-Difference method (DID) to study the impact of CTPP on carbon emissions in China. CTPP has been implemented since 2013. The DID method requires setting two sets of dummy variables. The first group was the treatment group and the control group, and the group that implemented the policy was the treatment group, with a value of 1. The control group was 0. The other group is the execution time of the policy, which is 0 before the policy is executed and 1 after the policy is executed. The interaction between the two groups is the Difference-in-Difference item (CTPP). The regression model is shown in following Equation (1).

(1) $${ln\_co2}_{it}=\delta_{0}+\beta_{i}{CTPP}_{it}+\beta_{r}X_{it}+\lambda_{i}+v_{t}+\varepsilon_{it}.$$

In Equation (1), ${\ln co2}_{it}$ is the explained variable, representing the carbon emission of i city in t years. ${CTPP}_{it}$ is the core explanatory variable, which is a 0–1 dummy variable if the implementation is 1 or 0. $\beta_{i}$ is the coefficient of CTPP in this paper. If the $\beta_{i}$ is significantly negative, it shows that CTPP is helpful in reducing carbon emissions.$X_{it}$ is the control variable at the city level. $\lambda_{i}$ is the city-fixed effect. $v_{t}$ is the time-fixed effect.

## 5. Empirical Results and Test

### 5.1. Benchmark Regression Results

Stepwise regression analysis uses the DID model to calculate the effect of the CTPP on carbon emissions. Table 2 presents the findings of the benchmark regression analysis. The influence of the CTPP on carbon emissions is examined independently in Column (1), and Columns (2) to (5) obtain reliable estimation outcomes by gradually introducing control variables based on Column (1). Column (5)’s CTPP coefficient, which is −0.0621 at the 1% confidence level, indicates that the CTPP significantly lowers China’s carbon emissions; the effect value of this policy is, therefore, 6.21%.

### 5.2. Parallel Trend Test

For a rise in the accuracy and conclusion’s dependability in the benchmark regression above, the parallel trend test is conducted in this subsection since the DID method’s underlying assumption is that the development trends of the treatment group, which consists of cities using CTPP, and the control group, which comprises cities not implementing CTPP, are parallel. This paper refers to Yang et al. [74]. Two dummy variables are added to Equation (1). The specific regression equation is as follows:

(2) $${ln\_co2}_{it}=\delta_{0}+\sum_{m=1}^{4}\delta_{m}{FirstCTPP}_{i,t-m}+{FirstCTPP}_{i,t}+\sum_{n=1}^{4}\delta_{n}{FirstCTPP}_{i,t+n}+\delta_{r}X_{it}+\lambda_{i}+v_{t}+\varepsilon_{it}$$

In Equation (2), ${FirstCTPP}_{I,t}$ is a dummy variable that refers to the first execution of CTPP. ${FirstCTPP}_{i,t}$ is 1 if the policy is implemented for the first time, and 0 otherwise. $\sum_{m=1}^{4}\delta_{m}{FirstCTPP}_{i,t-m}$ and $\sum_{n=1}^{4}\delta_{n}{FirstCTPP}_{i,t+n}$ are two dummy variables, respectively, four years before and four years after the policy.

**Figure 3:**
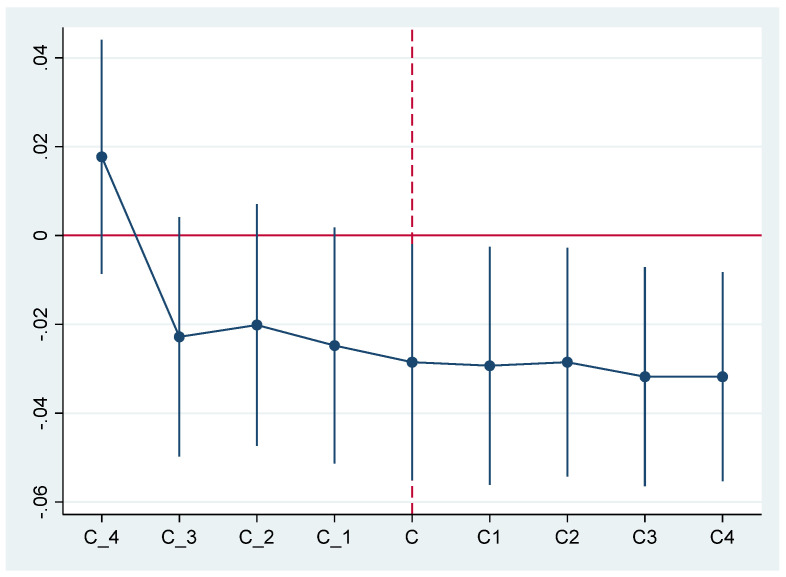
Regression results of the parallel trend test.

The results of the parallel trend test are shown in Figure 3. In the abscissa of Figure 3, c is the time of the first implementation of the policy. c1-c4 and c_1-c_4, respectively, represent the four years before and four years after the policy. The significance before and after CTPP is the opposite. The effect is significant after implementation but not before implementation. These test results meet the requirement of a parallel trend test. Therefore, the parallel trend test enhances the reliability of the baseline regression conclusion.

## 6. Robustness Test

### 6.1. Endogeneity Treatment

The ventilation coefficient is used in this article as an instrumental variable to assess potential endogeneity, taking into account endogeneity issues such as reverse causation or other omissions of other key variables.

In this paper, the Ventilation Coefficient (*ln_venti*) of cities was chosen as the instrumental variable of environmental policy variables with reference to Hering and Poncet [75]. The Ventilation Coefficient is considered the determinant of the diffusion rate of air pollution in standard box models of air pollution [76]. In the case of a particular total carbon emission, the smaller the cities’ Ventilation Coefficient is, the greater the air pollution concentration is monitored. Therefore, the government is likely to raise the bar for environmental oversight, and the city is more likely to be selected as the city for CTPP implementation, which satisfies the correlation hypothesis. In addition, since Ventilation Coefficient is determined by large-scale weather systems, there is no other action mechanism between the Ventilation Coefficient and carbon emission. Therefore, as an instrumental variable of CTPP, the ventilation coefficient satisfies the exogeneity hypothesis.

Table 3 displays the outcomes of endogenic processing. In Column (2), the ventilation coefficient (*ln_venti*) was the explanatory variable and CTPP is the explained variable, and the regression coefficient was −0.2973, and *p* < 0.01. This means that the smaller the ventilation factor, the more likely it is to be selected as a CTPP city.

The CTPP’s coefficient is −3.5167, which is still strongly negative according to Column (1) of Table 3. The F statistic is greater than 10 at 29.965. The Ventilation Coefficient (*ln_venti*) is a valid instrumental variable as a result. This suggests that even after the endogeneity test, the baseline regression results in this work are still accurate.

### 6.2. PSM-DID Test

#### 6.2.1. PSM Process

To test possible sample bias, Propensity Score Matching (PSM) was used for processing, followed by DID regression analysis. The outcome of the PSM method is to make the policy the only factor that distinguishes cities that adopt policies from those that do not. Propensity score values are obtained by Logit regression on the CTPP dummy variable using one-to-one matching with replacement. Matches are not tied, and if the propensity score is the same, the selection is sorted according to the data. The most important characteristic variables of matching are the Level of Opening-up (*ln_fdigdp*), Investment in Science and Technology (*ln_sciep*), Investment in Education (*ln_edue*), Economic Status (*ln_gdppop*), Information Status (*ln_internetp*), Population Density (*ln_popden*), Energy Consumption (*ln_gasp*, *ln_liqgasp*, *ln_elecp*).

**Figure 4:**
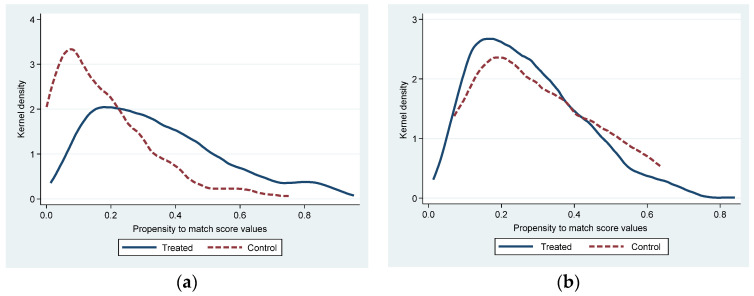
Common values before and after PSM treatment (2015). (**a**) Kernel density before PSM, (**b**) Kernel density after PSM.

The common value test and the matching balance test were used to evaluate the impact of PSM therapy. Take 2015, the middle of the policy’s implementation. The value zones of the treatment and the control group overlapped before matching, as seen in Figure 4a, demonstrating that the assumption of common value was met.

**Figure 5:**
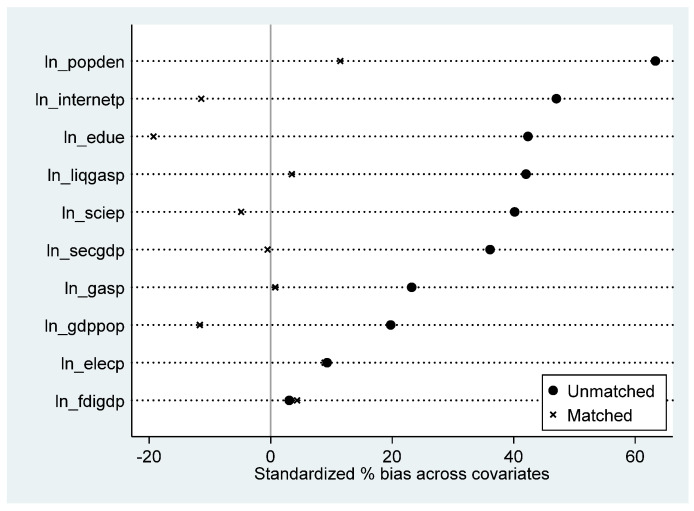
Standardized deviation of variables before and after PSM treatment (2015).

After PSM, the sample distribution of cities with and without policy implementation tends to be significantly consistent (See Figure 4b). The absolute values of standard deviations after PSM treatment were all less than 20% (see Figure 5 and Table 4). These matching results are valid [77], and the results meet the requirements of the matching balance test. All P-values in Table 3 exceed 0.1, indicating that the two types of variables are indifferent, so the results of the PSM are valid.

#### 6.2.2. PSM-DID Regression Results

The data were first processed using the Propensity Score Matching (PSM) approach, and then regression analysis was performed using the DID method. The results of stepwise regression using the DID technique are displayed in Table 5. With a confidence level of 1%, the coefficients of the Carbon Trading Pilot Policy (CTPP) are all significantly negative from Column (1) to Column (5), showing that the policy’s adoption significantly decreased carbon emissions. This indicates that there is no sample selection bias in baseline regression. These outcomes are in line with the baseline regression and once more support its results.

### 6.3. Variable Substitution, Lag Phase, and Time–Bandwidth

In order to test the benchmark regression’s reliability, the robustness test is further conducted from the following aspects: explanatory variable replacement, explanatory variable lagging one period, and changing the time window of regression.

First, this paper replaces the explanatory variable, namely the CTPP, with the representation of the Proportion of Environmental Statements (PES) in the Government Work Report. The higher the ratio, the more stringent the local government is on environmental issues, including carbon emissions. Second, considering the hysteresis of policy implementation in the benchmark regression, a robustness test was conducted for the CTPP with a lag of one year (*L1_CTPP*). Finally, to examine the impact of policy implementation time on carbon emissions, 2003–2017, 2003–2018, and 2003–2019 were selected as regression time ranges.

The Proportion of Environmental Statements (PES) in the Government Work Report is used as the explanatory variable in Column (1) of Table 6 instead. *PES*’s coefficient is −0.0693, with a confidence level of 1%. This shows a negative correlation between the importance of environmental protection and carbon emissions in the government work report.

Column (2) is the explanatory variable CTPP lagged by one year (*L1_CTPP*), and the coefficient of *L1_CTPP* is −0.0618, with a confidence level of 1%. This demonstrates the robustness of the baseline regression result.

Columns (3) to (5) represent the impact of the CTPP on carbon emissions during 2003–2017, 2003–2018, and 2003–2019 respectively, and their coefficients are −0.0586, −0.0594, and −0.0618, respectively, with a confidence level of 1%. The influence increases gradually with the coefficient, indicating that the impact of CTPP increases gradually with the increase in the implementation time.

### 6.4. Excluding Other Policy Interference

The repercussions of the CTPP on carbon emissions may be related to other environmental protection policies, which may be the direct or combined effect of other environmental protection policies. Other environmental policies, such as Low-carbon Cities and Smart Cities Policy, may also have carbon-reducing effects.

(1) Low-carbon Cities Policy (only one prefecture-level city that implemented the policy was deleted. In addition, Sanya city is repeated with the second list of cities, Yuxi city is repeated with the first list of cities, Ankang city is repeated with the first list of cities, and the duplicate prefecture-level cities are deleted. The sample in Smart Cities Policy is also treated). The Chinese government has proposed this development strategy as a proactive response to climate change and to encourage low-carbon development. The policy refers to the three batches of cities announced by relevant departments of the Chinese government from 2010 to 2017. The first batch was announced on 19 July 2010, involving 72 prefecture-level cities (Source: https://www.ndrc.gov.cn/xxgk/zcfb/tz/201008/t20100810_964674.html?code=&state=123, accessed on 1 November 2022). The second batch was announced on 26 November 2012, adding 24 prefecture-level cities (Source: http://gongyi.sina.com.cn/greenlife/2012-12-04/095739489.html, accessed on 1 November 2022). The third batch, announced on 7 Jan 2017, consists of 27 prefecture-level cities (Source: https://www.ndrc.gov.cn/xxgk/zcfb/tz/201701/t20170124_962888.html?code=&state=123, accessed on 1 November 2022).

(2) Smart Cities Policy. The Smart Cities Policy is the advanced development stage of urban digitalization, which promotes smart industry clusters and expands the application ecological scenarios of clean industries. Smart Cities Policy can encourage green and low-carbon development [78].

The first group of 90 “Smart Cities” in China, comprising 37 prefecture-level cities, was unveiled in December 2012 (Source: https://www.mohurd.gov.cn/xinwen/jsyw/201301/20130131_221676.html, accessed on 1 November 2022). In May 2013, 83 cities and districts, 20 counties or towns, and 9 cities and districts made up the second batch of “Smart Cities”, which was enlarged from the initial batch of pilot cities in 2012 (Source: https://www.mohurd.gov.cn/xinwen/gzdt/201308/20130808_214670.html, accessed on 1 November 2022). On 7 April 2014, the third batch of China’s Smart Cities list, which included 97 cities, counties, or districts, was made public (Source: https://www.mohurd.gov.cn/xinwen/gzdt/201504/20150414_220664.html, accessed on 1 November 2022).

Table 7 shows the above two kinds of policy regression results. The CTPP coefficient is obviously considered negative in the two types of policy samples, and the impact of this policy is stronger in low-carbon cities than in non-low-carbon cities and in smart cities than in non-smart cities. These findings show that CTPP significantly contributes to lowering carbon emissions. The reduction in carbon emissions is also related to Low-carbon Cities and Smart Cities Policies. Still, there is a possibility of the combined effect of these two kinds of policies.

## 7. Mechanism Test

### 7.1. Mechanism Test Steps

This section tests the three mechanism hypotheses. According to the mechanism analysis, CTPP promotes carbon reduction through Green Consumption Transformation (GCT), Eco-efficiency (EE), and Industrial Structure Upgrading (ISU). The method mechanism of Judd and Kenny [79] and Baron and Kenny [80] is used for reference to test.

First, the regression between CTPP and carbon emissions was examined. The test is passed if the CTPP coefficient is significant and conforms to theoretical expectations. Obviously, this step has been performed and passed in the baseline regression.

Second, CTPP conducted regression analysis on GCT, EE, and ISU, respectively. If the CTPP coefficient was significant, we continued the analysis. Otherwise, we would have stopped further analysis.

Third, the three mechanisms were placed in the regression equation together with CTPP. If the coefficient of CTPP is non-significant, it signifies complete mediation. If the CTPP coefficient is still significant, but the significance becomes smaller, or the coefficient’s absolute value decreases, it indicates that the mediation impact exists. The following is a model of the three steps above.

The regression model of the first step is the same as regression Equation (1).

The second step is the following regression models:

(3) $${GCT}_{it}\left(EE,ISU\right)=\delta_{0}+\alpha_{i}{CTPP}_{it}+\beta_{r}X_{it}+\lambda_{i}+v_{t}+\varepsilon_{it}.$$

The third step is the regression models:

(4) $${ln\_co2}_{it}=\delta_{0}+\gamma_{i}{CTPP}_{it}+\theta_{i}{GCT}_{it}\left(EE,ISU\right)+\beta_{r}X_{it}+\lambda_{i}+v_{t}+\varepsilon_{it}.$$

### 7.2. Construction of Mechanism Variables

Green Consumption Transformation (GCT). Jing et al. believe that the development of public transportation helps to lower carbon emissions and optimize the structure of energy usage [81]. For reference to the study, the percentage of public transportation to all public transportation and taxis is known as the GCT.

Industrial structure Upgrading (ISU). Guo and Peng believe that industrial structure change has an impact on green total factor productivity (GTFP) [82]. Therefore, the percentage of secondary industry and tertiary industry’s output value serves as an indicator of ISU.

Ecological efficiency (EE). In accordance with the approach by Yang et al., the eco-efficiency index is created using Data Envelopment Analysis (DEA) [83]. We adopt the Super-efficiency Slacks-Based-Measure (Super-SBM) with the output-oriented and constant return to scale to calculate the Ecological Efficiency (EE). Multiple cases of efficiency units can be distinguished by the Super-SBM model. Input variables and output variables are required for the measurement of Ecological Efficiency (EE), among which output includes desirable output and undesirable output. Inputs are capital and labor. Capital includes land and net fixed capital. Labor is represented by the end-of-year population count. GDP is the desired output. Industrial sulfur dioxide, industrial wastewater, and industrial carbon dioxide are undesired outputs.

### 7.3. Regression Results of Mechanism Test

In Columns (1), (2), and (3) of Table 8, the mediating effect of green consumption transformation (GCT) is put to the test. According to the regression with the benchmark in Column (1) of Table 8, it is evident that the test is passed by the first step. The Carbon Trading Pilot Policy (CTPP) coefficient in Column (2) of Table 8′s is 0.0362, which, at a 1% degree of confidence, is significantly positive, indicating that CTPP promotes GCT. Therefore, the second step test is passed. The GCT coefficient in Column (3) of Table 8 is −0.1286, which is markedly negative and shows that GCT can promote carbon emission reduction (ln_co_2_). The CTPP coefficient in Table 8′s Column (3) is −0.0575, which is lower than the CTPP value in Column (1), and proves that the test is passed by the third step. Therefore, the mediating effect of GCT is significant. That is, GCT is the intermediary mechanism of CTPP.

Table 8′s Columns (1), (2), and (3) examine the role of Green Consumption Transition (GCT) as a mediator between the three steps. Since Column (1) in Table 8 represents the same regression as the baseline, it is evident that the first step passes the test. In Table 8′s Column (2), the Carbon Trading Pilot Policy (CTPP) coefficient is 0.0362, and the confidence level is 1%, indicating that CTPP promotes GCT. Consequently, the second step is successful. In Table 8, Column (3) shows that the GCT coefficient is −0.1286, and the confidence level is 1%, demonstrating that GCT contributes to carbon emission reduction (ln_co_2_). Observe the CTPP coefficient and significance in Column (3) of Table 8′s, the degree of confidence is 1%, but the CTPP coefficient is smaller than that of Column (1). The third step passes the test. Therefore, the mediating effect of GCT is significant. In other words, GCT is the mediation mechanism of CTPP.

Similarly, by testing the mediating effect of Ecological Efficiency (EE) and Industrial Structures Upgrading (ISU), respectively, it is easy to determine that EE and ISU are the mediating mechanisms of CTPP carbon emission reduction.

Conclusion: Through GCT, EE, and ISU, the CTPP can promote the reduction in carbon emissions.

### 7.4. Mechanism Contribution Decomposition

Based on the investigation of Heckman et al. [84] and Gelbach [85], combined with regression Equations (1), (3) and (4), the contribution of carbon emission reduction to Green Consumption Transformation (GCT), Ecological Efficiency (EE) improvement, and Industrial Structures Upgrading (ISU) can be calculated by $\theta_{i}$ × $\alpha_{i}/\beta_{i}$. $\beta_{i}$ is the coefficient of CTPP in regression Equation (1). $\alpha_{i}$ is the coefficient of CTPP in regression Equation (3). $\theta_{i}$ is the coefficient of the GCT (EE, ISU) of regression Equation (4).

The contribution of the GCT (EE, ISU) is shown in Table 9. The contribution of GCT is 7.50%, the contribution of EE is 2.25%, and the contribution of ISU is 1.66%. This shows that the CTPP at the current stage relies more on promoting GCT and improving EE to achieve carbon emission reduction.

## 8. Heterogeneity Analysis

### 8.1. Regional Heterogeneity

The regional heterogeneity of the CTPP’s influence on carbon emissions will be tested in this section. Eastern, central, and western samples were separated into three groups [83]. There are 285 cities altogether, 101 of them in the east, 100 in the middle, and 84 in the west.

Table 10′s Columns (1) through (3), respectively, show the findings of the regression for the eastern, central, and western regions. The center region’s CTPP coefficient is the highest, indicating that CTPP has the greatest influence in the central cities. The following could be the cause: The central cities are undertaking the industrial transfer of the eastern cities, and the proportion of high-consumption and high-emission industries is larger. Hence, the marginal effect of policy implementation is greater.

### 8.2. Administrative Heterogeneity

Cities in China are categorized into municipalities, provincial capitals, and particular economic zone cities. They may have differences in resource endowment and industrial structure, economic strength, and public attitude toward the environment. Therefore, depending on the administrative level of city changes, the CTPP may have different effects on carbon emissions.

According to the classification method proposed by Yang et al. [83], cities are separated into core and peripheral cities. The core cities mainly include the provincial capital city, deputy provincial capital city, particular economic zone cities, and separately listed cities. The peripheral cities are common prefecture-level cities. Therefore, this article separates 285 cities into core and peripheral cities, 36 and 249, respectively.

Table 11 shows the regression results in core and peripheral cities, respectively. As demonstrated by Table 11, both cities’ carbon emissions are significantly impacted negatively by CTPP. In the peripheral cities, the confidence level and coefficient of CTPP are higher. The possible reasons are that CTPP facilitates the transfer of energy-intensive and polluting industries from core cities to peripheral cities or increases in energy efficiency and a shift to green consumption. The marginal impact of CTPP on carbon emissions reduction in peripheral cities is, therefore, larger.

## 9. Discussion

In 2013, Shenzhen took the lead in launching CTPP in China. Later, six provinces, and cities including Beijing, Shanghai and Guangdong set up CTPP. In 2016, Sichuan and Fujian provinces joined CTPP. These provinces and cities are the sample space range of this paper. China’s carbon trading policy market has achieved remarkable results. Like most of the literature, the results of our study are consistent with the actual policy outcomes of CTPP in China.

However, at the academic level, the conclusion of the CTPP policy effect is not completely consistent with that of other scholars. The different performance is mainly reflected in the following aspects. First, the effect of carbon reduction is inconsistent. In this study, it is suggested that China’s CTPP reduces carbon emissions by 6.21%, while some studies by other scholars show that it reduces carbon emissions by 15.5% (Hu and Ren et al., 2020) [28], and 20.06% (Zhang and Shi et al., 2017) [24]. This may be related to different scholars’ research spaces, data, or model methods. Second, the transmission mechanism is inconsistent. This paper holds that the mechanism of CTPP to reduce carbon emissions is Green Consumption Transformation (GCT), improving Ecological Efficiency (EE), and promoting Industrial Structure Upgrading (ISU). Our study does not detect that technological innovation is the mediating mechanism of CTPP, which is consistent with the study of Xia and Li et al. (2020) [25], but Liu, Ma, and Xie (2020) [86] believe that technological innovation is the mediating mechanism. Third, heterogeneity analysis is inconsistent. In addition, to sample regression from east, central, and west in China, this paper also conducts subsample regression of core and peripheral cities, hoping to obtain more detailed conclusions.

The cause of the above differences may have the following several aspects. First, the sample selection space is different. Most of the previous studies were carried out at the provincial level, less at the prefecture-level city level. Second, data sources and index construction are different. Third, the measurement method is different. Some used the synthetic control method (Chen and Lin, 2021) [87], others used the data simulation method, but most papers used DID method for empirical research. DID is effective in testing policy impact. Fourth, the robustness test is different. Unlike the previous literature, in order to improve the reliability of the conclusions, our paper carries out a variety of robustness tests, such as parallel trend test, endogeneity treatment, and selection bias test by using PSM. However, the previous literature does not offer such a comprehensive analysis.

In general, more detailed data and more rigorous empirical analysis on the carbon reduction effect of CTPP are expected from future scholars.

## 10. Conclusions and Policy Enlightenment

### 10.1. Conclusions

Adopting environmental policy to reduce carbon emissions is a crucial measure of environmental governance. This study builds the balance panel data of 285 Chinese cities between 2003 and 2020, involving 5130 samples. This study uses the Difference-in-Difference (DID) method to explore the effect of the CTPP on carbon emissions reduction. The conclusion is as follows.

First, according to the findings, the CTPP implementation considerably lowers carbon emissions by 6.21%.

Second, through a series of tests, we determine that the conclusion is robust. (1) To test possible reverse causality and variable omission, the ventilation coefficient was selected as the instrumental variable of CTPP for the endogeneity test, and regression analysis showed that the conclusion was still valid. (2) The data were processed using Propensity Score Matching (PSM) and the DID method for regression analysis to evaluate for potential sample selection bias, and the result was still robust. (3) In addition, by replacing the explanatory variable, the explanatory variable lags one stage and changes the sample’s time–bandwidth. The conclusion are still consistent. (4) With the increase in time–bandwidth, the effect of CTPP gradually increases. The paper distinguishes other environmental policies, including the Low-carbon Cities Policy and Smart Cities Policy on carbon emissions. We report that the effect of CTPP in these two types of cities is more excellent, indicating that there is a superimposed effect of environmental policies.

Third, the mechanism test and analysis show that CTPP can reduce carbon emissions through three intermediary mechanisms: Green Consumption Transformation (GCT), Ecological Efficiency (EE), and Industrial Structure Upgrading (ISU). The further contribution decomposition shows that among the three mechanisms, the contribution of green consumption transformation is the largest, with a value of 7.5%, followed by ecological efficiency and industrial structure upgrading, with 2.25% and 1.66%, respectively.

Fourth, the heterogeneity analysis shows that CTPP has the biggest marginal impact on reducing carbon emissions in central cities and peripheral cities.

### 10.2. Enlightenments

According to the empirical research of this paper, we propose the following policy recommendations.

First, expand the scope of the CTPP and accelerate the establishment of a unified national carbon emission trading market. CTPP is conducive to promoting carbon reduction in cities and is worth promoting nationwide. China is the largest carbon emitter, and the successful implementation of CTPP has made great contributions to global greenhouse gas emission reduction.

Second, establish a reasonable carbon allocation system. We should guide enterprises and society to take an active role in carbon trading, increase the popularity of carbon trading, improve the market management regulatory system for carbon trading, and boost the effectiveness of environmental law enforcement, oversight, and governance.

Third, adopt a variety of carbon reduction measures and provide full play to the integrated role of policies. As consumers, we should vigorously advocate the concept of low-carbon consumption and low-carbon life. In terms of regional economic development, we should guide regional industrial structure upgrading and develop low-carbon industries. On the energy front, technological reform should be carried out to improve energy efficiency. In addition, cities’ carbon emission reduction should be considered in combination with Low-carbon City Policies and Smart Cities Policies to exert the effect of policy superposition.

Fourth, policy measures should pay attention to urban and regional heterogeneity. In order to better promote the construction of low-carbon cities and accelerate the construction of an ecologically friendly society, local governments must pay attention to the differences in CTPP in different regions and cities and consider adopting targeted policies.

## Figures and Tables

**Table 1:** Variable definitions and descriptive statistics.

Variables	Variable Definitions	N	Mean	SD	Min	Max
ln_co_2_	Carbon Emissions	5130	15.4135	0.9349	12.2563	18.2213
CTPP	Carbon Trading Pilot Policy	5130	0.0799	0.2712	0.0000	1.0000
ln_fdigdp	Level of Opening-up	5130	2.6598	1.3608	−3.8321	7.1891
ln_sciep	Per capita R&D investment	5130	3.4965	1.7995	−8.6364	9.4090
ln_edue	Education Input	5130	12.4786	1.1225	6.7262	16.2476
ln_gdppop	Economic Status	5130	1.0325	0.9054	−1.6654	3.9747
ln_internetp	Information Status	5130	6.8714	1.1316	2.0597	10.5088
ln_secgdp	Industrial Structure	5130	3.8169	0.2694	0.9783	4.5105
ln_popden	Population Density	5130	5.7268	0.9175	1.5475	8.9603
ln_gasp	Per Capita Natural Gas Supply	5130	2.8205	1.6227	0.0000	10.1570
ln_liqgasp	Per Capita Liquefied Gas Supply	5130	3.3438	1.3527	0.0000	8.7804
ln_elecp	Per Capita Electricity Consumption	5130	6.7202	1.4881	1.3464	11.8274
ln_venti	Ventilation Coefficient	5130	7.0840	0.3858	5.6723	8.2591
GCT	Green Consumption Transformation	5130	0.3260	0.1754	0.0102	0.9994
EE	Ecological Efficiency (EE)	5130	0.3399	0.2502	−2.8958	1.3261
ISU	Industrial Structure Upgrading	5130	10.4328	3.2601	1.9022	38.8399

Note: “ln” means the logarithm of the variables.

**Table 2:** Benchmark regression results.

	(1)	(2)	(3)	(4)	(5)
	ln_co_2_	ln_co_2_	ln_co_2_	ln_co_2_	ln_co_2_
CTPP	−0.0376 ***	−0.0638 ***	−0.0694 ***	−0.0619 ***	−0.0621 ***
	(0.0118)	(0.0145)	(0.0130)	(0.0126)	(0.0126)
ln_fdigdp		0.0011	0.0006	0.0008	0.0011
		(0.0037)	(0.0037)	(0.0035)	(0.0035)
ln_sciep		0.0116	−0.0056	−0.0058	−0.0072
		(0.0072)	(0.0051)	(0.0051)	(0.0050)
ln_edue		0.3057 ***	0.1645 ***	0.1598 ***	0.1580 ***
		(0.0165)	(0.0166)	(0.0178)	(0.0177)
ln_gdppop			0.2477 ***	0.2728 ***	0.2737 ***
			(0.0219)	(0.0256)	(0.0264)
ln_internetp			0.0199 ***	0.0202 ***	0.0198 ***
			(0.0066)	(0.0064)	(0.0065)
ln_secgdp				−0.1100 ***	−0.1072 ***
				(0.0375)	(0.0381)
ln_popden				−0.0693	−0.0714
				(0.0577)	(0.0577)
ln_gasp					0.0072 *
					(0.0040)
ln_liqgasp					−0.0058
					(0.0051)
ln_elecp					−0.0008
					(0.0053)
N	5130	5130	5130	5130	5130
adj. R^2^	0.474	0.866	0.887	0.890	0.890

Note: The symbols for 10%, 5%, and 1% levels of statistical significance are *, **, and ***, respectively.

**Table 3:** Two-stage regression results.

	(1)	(2)
	2SLS	First stage
variable	ln_co_2_	CTPP
CTPP	−3.5167 ***	
	(0.4787)	
ln_venti		−0.2973 ***
		(0.0546)
Control variables	YES	YES
N	5130	5130
adj. R^2^	0.091	0.230
F statistic		29.965

Note: The symbols for 1% levels of statistical significance is ***.

**Table 4:** Deviation and confidence level before and after PSM (2015).

Variable	Unmatched	Mean			%reduct	*t*-test	
	Matched	Treated	Control	%bias	|bias|	*t*	*p* > |t|
ln_fdigdp	U	2.6841	2.6478	3.1		0.20	0.840
	M	2.6465	2.5951	4.3	41.5	0.21	0.835
ln_sciep	U	4.8662	4.3759	40.2		3.07	0.000
	M	4.7538	4.8133	4.9	87.9	0.25	0.806
1n edue	U	13.4020	13.0740	42.3		3.15	0.000
	M	13.3420	13.4910	19.3	54.4	1.11	0.268
1n_gdppop	U	1.5941	1.4526	19.7		1.48	0.139
	M	1.5216	1.6051	11.7	40.9	0.60	0.547
1n_internetp	U	7.6395	7.3362	47.0		3.67	0.006
	M	7.5385	7.6123	11.4	75.7	0.62	0.538
ln_secgdp	U	3.8823	3.8019	36.1		2.39	0.018
	M	3.8765	3.8776	0.5	98.6	0.04	0.970
ln_popden	U	6.1571	5.6272	63.3		4.14	0.000
	M	6.0598	5.9641	11.4	81.9	0.71	0.481
ln_gasp	U	3.5108	3.1655	23.2		1.67	0.096
	M	3.3879	3.3770	0.7	96.8	0.04	0.968
ln_liqgasp	U	3.7366	3.1010	42.0		3.34	0.001
	M	3.5251	3.4726	3.5	91.7	0.20	0.845
ln_elecp	U	6.8566	6.7329	9.3		0.65	0.516
	M	6.7103	6.5922	8.9	4.6	0.45	0.657

**Table 5:** PSM-DID regression results.

	(1)	(2)	(3)	(4)	(5)
	ln_co_2_	ln_co_2_	ln_co_2_	ln_co_2_	ln_co_2_
CTPP	−0.0282 **	−0.0478 ***	−0.0587 ***	−0.0556 ***	−0.0562 ***
	(0.0129)	(0.0147)	(0.0131)	(0.0130)	(0.0128)
ln_fdigdp		−0.0023	−0.0031	−0.0026	−0.0027
		(0.0041)	(0.0041)	(0.0040)	(0.0040)
ln_sciep		0.0155 **	0.0009	0.0003	0.0011
		(0.0071)	(0.0057)	(0.0057)	(0.0056)
ln_edue		0.3027 ***	0.1682 ***	0.1701 ***	0.1660 ***
		(0.0159)	(0.0170)	(0.0184)	(0.0188)
ln_gdppop			0.2327 ***	0.2428 ***	0.2477 ***
			(0.0228)	(0.0249)	(0.0260)
ln_internetp			0.0164 **	0.0158 **	0.0166 **
			(0.0072)	(0.0071)	(0.0072)
ln_secgdp				−0.0473	−0.0423
				(0.0366)	(0.0371)
ln_popden				−0.0939	−0.0858
				(0.0945)	(0.0928)
ln_gasp					0.0076 *
					(0.0043)
ln_liqgasp					−0.0074
					(0.0055)
ln_elecp					−0.0059
					(0.0056)
N	4439	4439	4439	4439	4439
adj. R^2^	0.450	0.874	0.890	0.891	0.891

Note: The symbols for 10%, 5%, and 1% levels of statistical significance are *, **, and ***, respectively.

**Table 6:** Regression results of variable replacement lag phase and time–bandwidth.

	(1)	(2)	(3)	(4)	(5)
	Explanatory variable replace	Lagged one year	In 2003–2017	In 2003–2018	In 2003–2019
	ln_co_2_	ln_co_2_	ln_co_2_	ln_co_2_	ln_co_2_
PES	−0.0693 *				
	(0.0407)				
L1_CTPP		−0.0618 ***			
		(0.0119)			
CTPP			−0.0586 ***	−0.0594 ***	−0.0618 ***
			(0.0124)	(0.0124)	(0.0125)
Control variables	YES	YES	YES	YES	YES
N	5130	5130	4275	4560	4845
adj. R^2^	0.865	0.890	0.893	0.892	0.890

Note: The symbols for 10% and 1% levels of statistical significance are * and ***, respectively.

**Table 7:** Results of regression excluding other policy interference.

	(1)	(2)	(3)	(4)
	In Low-carbon cities	In Non-low-carbon cities	In Smart cities	In Non-smart cities
	ln_co_2_	ln_co_2_	ln_co_2_	ln_co_2_
CTPP	−0.0809 ***	−0.0721 ***	−0.0808 ***	−0.0589 ***
	(0.0183)	(0.0170)	(0.0221)	(0.0149)
Control variables	YES	YES	YES	YES
N	2250	2880	1674	3456
adj. R^2^	0.894	0.891	0.908	0.882

Note: The symbols for 1% levels of statistical significance is ***.

**Table 8:** Regression results of mediating mechanism test.

	(1)	(2)	(3)	(4)	(5)	(6)	(7)
	ln_co_2_	GCT	ln_co_2_	EE	ln_co_2_	ISU	ln_co_2_
CTPP	−0.0621 ***	0.0362 ***	−0.0575 ***	0.0208 **	−0.0607 ***	0.1395 ***	−0.0450 ***
	(0.0126)	(0.0139)	(0.0126)	(0.0105)	(0.0066)	(0.0464)	(0.0061)
GCT			−0.1286 ***				
			(0.0136)				
EE					−0.0673 ***		
					(0.0090)		
ISU							−0.0074 ***
							(0.0019)
Control variables	YES	YES	YES	YES	YES	YES	YES
N	5130	5130	5130	5130	5130	5130	5130
adj. R^2^	0.890	0.132	0.894	0.395	0.885	0.894	0.901

Note: The symbols for 5%, and 1% levels of statistical significance are **, and ***, respectively.

**Table 9:** Mechanism contribution decomposition.

Time	Mechanism	$\theta_{i}$	$\alpha_{i}$	$\beta_{i}$	$\mathrm{Contribution}\;(\theta_{i}\;\times \;\alpha_{i}/\beta_{i})$
2003–2020	Green Consumption Transformation (GCT)	−0.1286 ***	0.0362 ***	−0.0621 ***	7.50%
	Ecological Efficiency (EE)	−0.0673 ***	0.0208 **	−0.0621 ***	2.25%
	Industrial Structure Upgrading (ISU)	−0.0074 ***	0.1395 ***	−0.0621 ***	1.66%

Note: The symbols for 5%, and 1% levels of statistical significance are **, and ***, respectively.

**Table 10:** Regression results by region.

	(1)	(2)	(3)
	In Eastern Cities	In Central Cities	In Western Cities
	ln_co_2_	ln_co_2_	ln_co_2_
CTPP	−0.0762 ***	−0.0859 ***	−0.0701 ***
	(0.0184)	(0.0256)	(0.0251)
Control variables	YES	YES	YES
N	1818	1800	1512
adj. R^2^	0.908	0.893	0.891

Note: The symbols for 1% levels of statistical significance is ***.

**Table 11:** Regression results from city level.

	(1)	(2)
	In Core Cities	In Peripheral Cities
variable	ln_co_2_	ln_co_2_
CTPP	−0.0493 **	−0.0777 ***
	(0.0240)	(0.0137)
Control variables	YES	YES
N	648	4482
adj. R^2^	0.911	0.885

Note: The symbols for 5%, and 1% levels of statistical significance are **, and ***, respectively.

## Data Availability

Not applicable.
